# Nodular hidradenoma mimicking amelanotic melanoma: A dermoscopic pitfall

**DOI:** 10.1016/j.jdcr.2026.07.025

**Published:** 2026-07-21

**Authors:** Juanita Montealegre-Montero, Sebastián Guerrero-López, Karen Viviana Arévalo-Mendes, Isabela Dorado-Caycedo

**Affiliations:** a Dermatology Resident - School of Medicine - Fundación Universitaria Sanitas, Bogotá, Colombia; b Dermatology Resident - School of Medicine - Pontificia Universidad Javeriana, Bogotá, Colombia; c Dermatologist – Centros Médicos Virrey Solis, Bogotá, Colombia; d School of Medicine, Fundación Universitaria Sanitas, Bogotá, Colombia; e Dermatologist – Hospital Universitario La Samaritana, Bogotá, Colombia

**Keywords:** adnexal tumor, amelanotic melanoma, dermoscopy, eccrine hidradenoma, nodular hidradenoma, sweat gland neoplasm

## Clinical presentation

**Fig 1:**
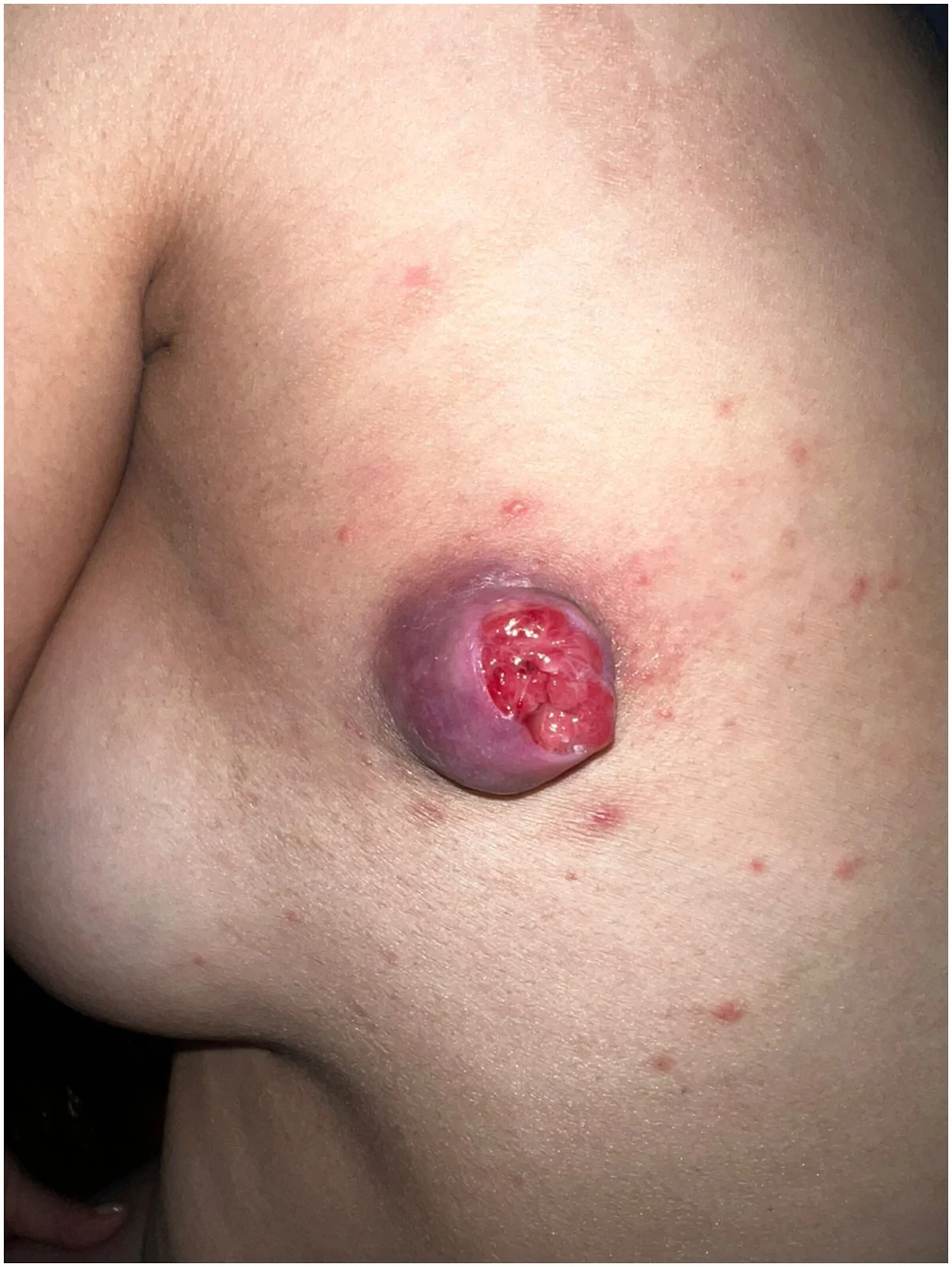
Erythematous-violaceous exophytic, dome-shaped and indurated tumor with central ulceration, purple-bluish hue, and serous discharge, measuring 30 × 30 × 28 mm. Associated with peripheral erythematous papules.

A 37-year-old woman presented with a 1-year history of a progressively enlarging painful nodule along the left posterior axillary line. Examination revealed an erythematous-violaceous exophytic, dome-shaped and indurated tumor with central ulceration, purple-bluish hue, and serous discharge, measuring 30 × 30 × 28 mm associated with peripheral erythematous papules (Fig 1). A nontender adenopathy was palpated on the left axilla.

## Dermoscopic appearance

**Fig 2:**
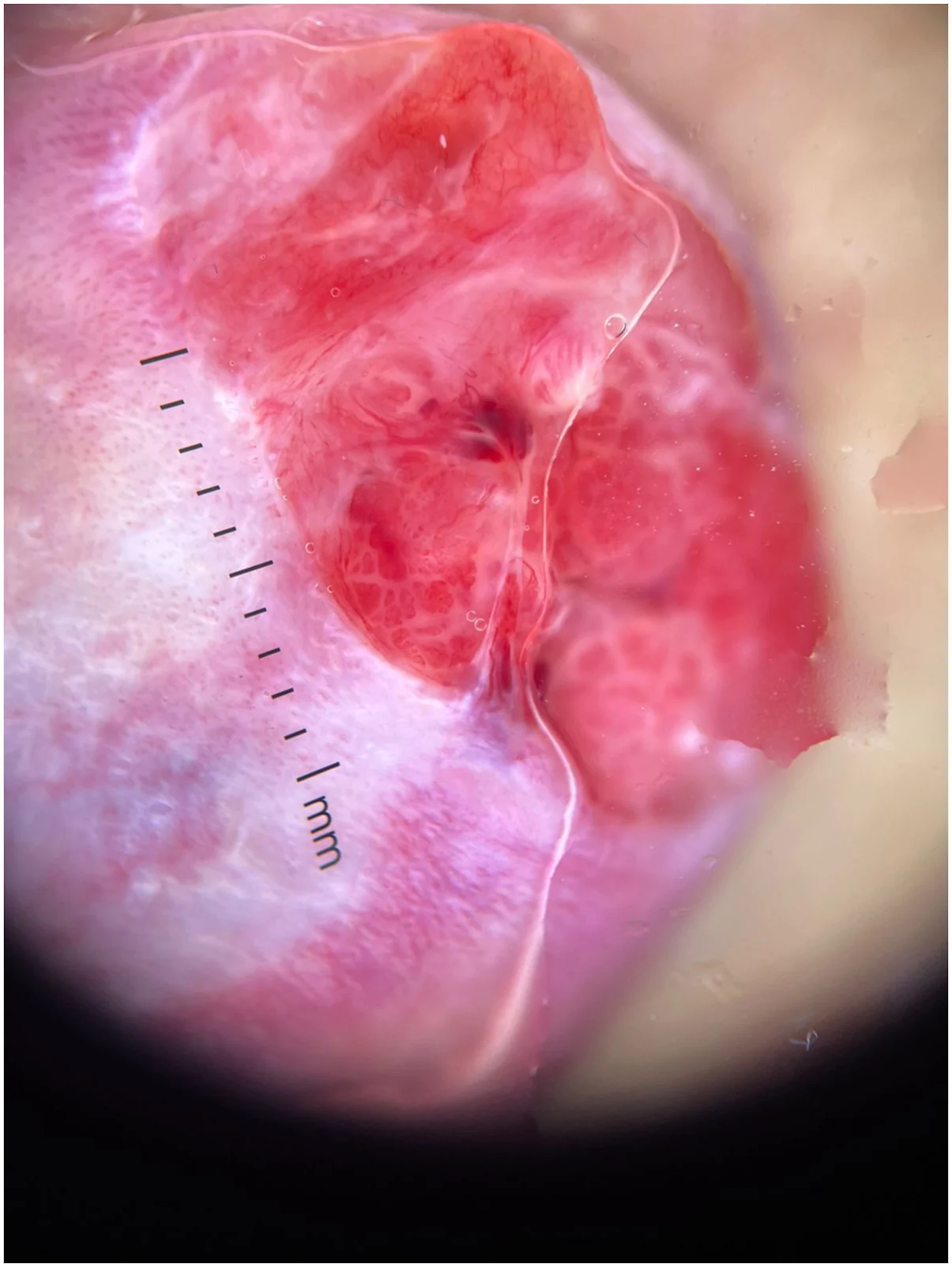
Dermoscopy (DL5 polarized, contact type) showing well-defined pink-white and bluish-pink homogeneous areas, white structures, polymorphous vessels and ulceration.

Dermoscopy showed well-defined pink-white and bluish-pink homogeneous areas, white structures, a symmetric arrangement of polymorphous vessels and central ulceration (Fig 2). Given these clinical and dermoscopic findings, amelanotic melanoma, adnexal tumors, and cutaneous metastases were considered in the differential diagnosis. A breast ultrasound, chest computed tomography, and soft-tissue ultrasound were performed revealing only 2 residual calcified lymph nodes in the right hilar region, compatible with postinflammatory residual changes.

## Histologic diagnosis

**Fig 3:**
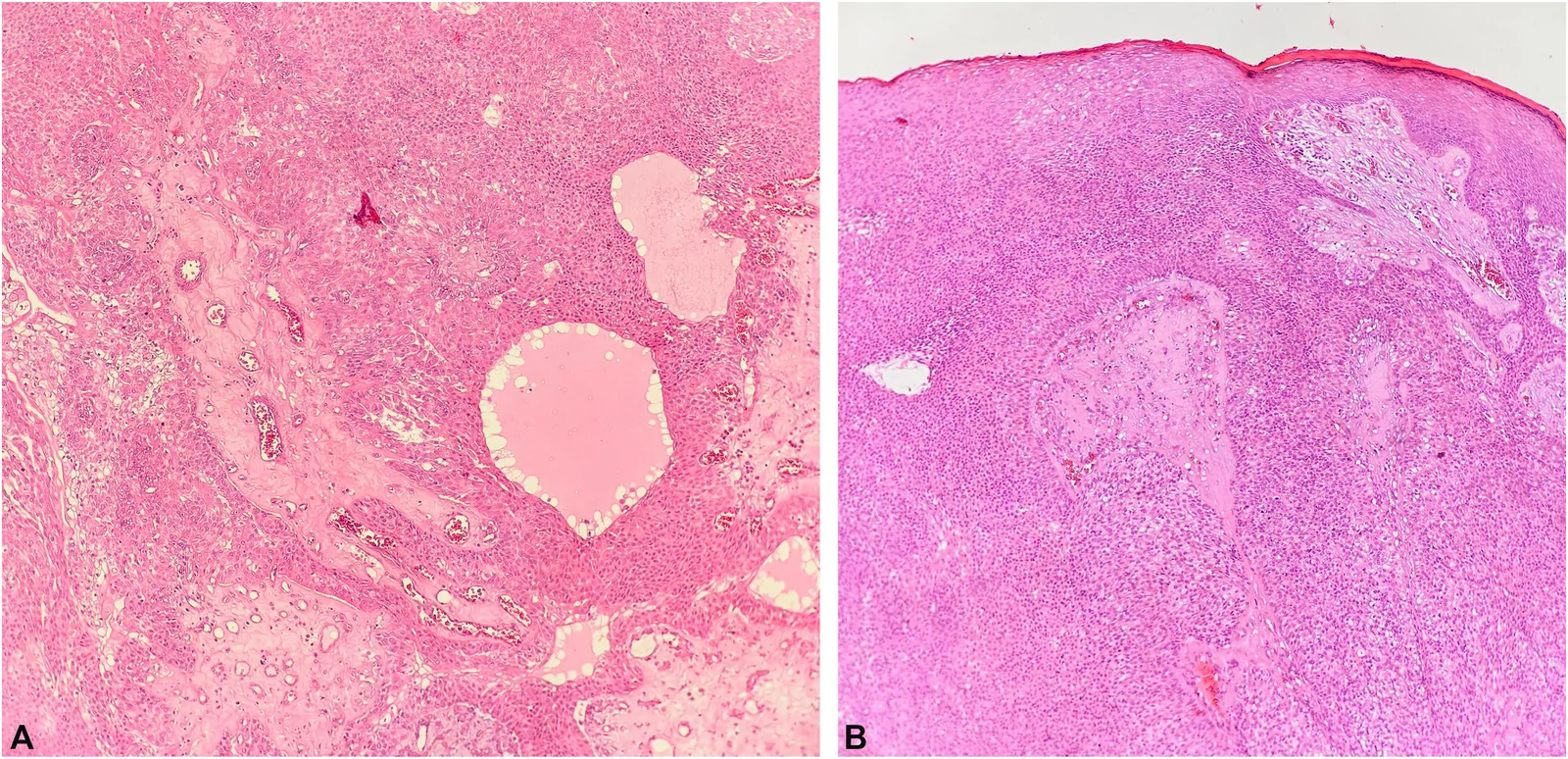
Histopathology of nodular hidradenoma (hematoxylin-eosin stain). **A,** Well-circumscribed solid and cystic dermal neoplasm with hyalinized stroma and congested capillaries. **B,** Panoramic view showing the well-circumscribed dermal tumor with solid nests and cystic-ductal structures; no significant cytologic atypia is identified.

An excisional biopsy was performed. Histopathologic examination revealed a well-circumscribed dermal neoplasm composed of polygonal basaloid cells arranged in solid and cystic nests with focal ductal differentiation, embedded within a hyalinized stroma with congested capillaries (Fig 3, *A* and *B*). No significant cytologic atypia, necrosis, or infiltrative growth was identified. These findings established the diagnosis of nodular hidradenoma.

Key message

Nodular hidradenoma should be included in the dermoscopic differential diagnosis of amelanotic tumors: its homogeneous white-pink and bluish-pink areas and white structures, reflecting the underlying hyalinized stroma and intratumoral cysts together with polymorphous vessels and ulceration, may closely mimic amelanotic melanoma. Histopathologic examination remains essential for definitive diagnosis and to avoid overtreatment.^1^^,^^2^

## Conflicts of interest

None disclosed.
